# Combination of BeGraft and Solaris Stent Grafts for the Covered Endovascular Reconstruction of Aortic Bifurcation—BS-CERAB Technique

**DOI:** 10.3390/jcm13071925

**Published:** 2024-03-26

**Authors:** Enrique M. San Norberto, Álvaro Revilla, José A. Brizuela, Isabel Del Blanco, Ángel Flores, James H. Taylor

**Affiliations:** 1 Department of Angiology and Vascular Surgery, Valladolid University Hospital, 47003 Valladolid, Spain; arevilla@saludcastillayleon.es (Á.R.); jabrizuela@saludcastillayleon.es (J.A.B.); iblancoa@saludcastillayleon.es (I.D.B.); 2 Department of Angiology and Vascular Surgery, Toledo University Hospital, 45007 Toledo, Spain; aflores@sescam.jccm.es; 3 Department of Angiology and Vascular Surgery, Valencia General University Hospital, 46014 Valencia, Spain; ira_tenax99@hotmail.com

**Keywords:** aortoiliac occlusive disease, covered stent, percutaneous transluminal angioplasty, stenting, iliac artery stenosis

## Abstract

**Background**: This study examines the impact of the use of the combination of BeGraft and Solaris stent grafts on the outcomes during the covered endovascular reconstruction of aortic bifurcation (BS-CERAB) technique and extension to the iliac arteries. **Methods**: Consecutive patients with aortoiliac occlusive disease who underwent endovascular treatment using BS-CERAB between January 2020 and December 2023 were included. Patient demographics, symptoms, lesion characteristics, and procedural and follow-up details were collected and analyzed. Perioperative complications and reinterventions were also identified. **Results**: A total of 42 patients met the inclusion criteria (32 men, 76.2%, median age 72 years, range 59–85). Indications for treatment were intermittent claudication (42.9%) and critical limb ischemia (57.1%). Procedure success was achieved in all cases. The median patient follow-up time was 14 months (1–36). One patient died at a 10-month follow-up due to lung cancer. The mean pre-operative ABI increased from 0.37 ± 0.19 before intervention to 0.71 ± 1.23 post-operatively at 12 months (*p* = 0.037). The estimated primary patency rates at 3, 6, and 12 months were 90.5%, 85.7%, and 81.0% and primary assisted patency rates were 90.5%, 90.5%, and 85.7%, respectively. Secondary patency was 95.2% at 3 and 6 months and 90.5% at a 12-month follow-up. Active cancer (*p* = 0.023, OR 2.12 95%CI 1.14–3.25) was a risk factor for restenosis. **Conclusions**: This mid-term experience shows that the CERAB technique using the combination of BeGraft and Solaris stents grafts, for the endovascular treatment of severe aortoiliac atherosclerotic disease, may allow an effective reconstruction of the aortic bifurcation and iliac arteries related to high-patency and lower-reintervention rates.

## 1. Introduction

The covered endovascular reconstruction of aortic bifurcation (CERAB) technique described by Goverde et al. [1] in 2013 was introduced to reconstruct the aortic bifurcation in a more anatomical and physiological way. In this way, a balloon-expandable covered stent is deliberately expanded over 15 to 20 mm above the aortic bifurcation. This stent is then distally adapted to the aortic wall with a bigger balloon, thereby resulting in a cone-shaped stent. The other inwardly curved stent is then placed into the distal conic segment and simultaneously inflated in such a way that it makes a tight connection with the aortic stent as if they were molded. This creates a new bifurcation [2]. This minimally invasive endovascular technique was developed using covered stents in order to imitate the anatomical configuration of the aortic bifurcation and had the aim of avoiding the disadvantages caused by geometric factors, such as radial and protrusion mismatches and stent conformation.

Several factors may play a role in patency rates of the endovascular treatment of the steno-occlusive disease of the aortic bifurcation, such as hemodynamic forces, neointimal hyperplasia, differences in outflow disease, residual stenosis after endovascular treatment, or the total atherosclerotic burden [3]. The covered stents form a mechanical barrier compared to bare stents, excluding the plaque and endothelium, thereby limiting intimal hyperplasia. In addition, the decreased risk of aortic or iliac rupture during dilatation when using a covered stent could allow inflation to a higher pressure, which may also explain the patency gain. Another theoretical advantage is related to the protrusion of the bare stents into the distal aortic lumen, creating a new flow divider within the opposing stents, the stent overlap, and other geometric determinants. Thus, the positioning of the stents and the difference in radial dimension between the lumina of the aortic stents and the aortic lumen (radial mismatch) may generate significant flow turbulences and thrombus formation [4]. Therefore, the radial mismatch effect on patency rates of covered stents may vary from that of bare metal stents and their outcomes may differ accordingly because the covering material will not allow flow through the radial mismatch area between the stented lumen and the real arterial lumen. Obviously, this may reduce the occurrence of stasis, recirculation, and turbulence of blood. Accordingly, these factors may influence the shear stress and, therefore, leave bare metal stents without protection, increasing the risk of thrombosis and in-stent restenosis caused by intimal hyperplasia. Further flow studies could provide additional information to elucidate this complex mechanism of how flow is affected by radial stent mismatch in different configurations and their patency rates. Further, the iliac stents extended into the aorta can create free-floating ends in the distal aorta that might distort the fluid flow around these elements, causing non-targeted flow perturbations. Moreover, this technique presents favorable outcomes during the lesions’ recanalization, preventing complications like perforation, embolization, or flow-limiting dissection [5].

Covered stents have been reserved for arteriovenous fistulas, iliac aneurysms, or iatrogenic perforations. However, recent studies have provided encouraging results of covered stents compared with bare metal stents for aortoiliac lesions. Several covered stents have been used with technical success for CERAB. Most of these stents are made of stainless steel (Atrium Advanta V12, Gore Viabahn VBX, Bard LifeStream) whereas others are made of cobalt chromium (Bentley BeGraft Aortic, Hechingen, Germany). The design of these stents differs significantly and the way the covering material is applied differs from polytetrafluoroethylene on both sides of the stent (Advanta, VBX, LifeStream) or an external layer only (BeGraft) [5]. Simultaneously to the introduction of the CERAB technique, the use of the AFX bifurcation endograft (Endologix) has been described for the treatment of aortoiliac complex steno-occlusive lesions.

The aim of the current study was to report on the short- and medium-term outcomes of the CERAB technique for extensive aortoiliac occlusive disease, performed by the combination of BeGraft and Solaris stent grafts (BS-CERAB).

## 2. Materials and Methods

### 2.1. Study Design

This retrospective cohort study included patients who underwent endovascular treatment for severe atherosclerotic steno-occlusive aortoiliac lesions between January 2020 and December 2023 in the Valladolid University Hospital and the Toledo University Hospital, two Spanish tertiary centers. For this analysis, only those treated with the BeGraft and Solaris stent graft combination were included in the study cohort. This study, which received no financial support from industry, was performed in agreement with the Declaration of Helsinki and was approved by the institutional review boards. In accordance with institutional and local regulatory policies, this retrospective review of de-identified procedural and follow-up data was exempt from informed consent. This study was registered on clinicaltrial.gov under the identifier NCT06012123. The study design and this manuscript were adapted to the STROBE guidelines for observational studies (see Supplementary Table S1).

Inclusion criteria were patients with lifestyle-limiting intermittent claudication, ischemic rest pain, ischemic ulcers, or gangrene (Rutherford Class 3 to 6) who presented steno-occlusive disease of the aortic bifurcation with lesions TASC-II Type D, undergoing endovascular treatment by the CERAB technique with the combination of BeGraft and Solaris stent grafts. Exclusion criteria were patients who could not receive antiplatelet or anticoagulation therapies. Other exclusion criteria were patients with a concomitant aneurysm of the aorta, acute thrombus, unsalvageable limb, or very limited life expectancy.

During the 48-month period, 271 patients with lesions in the aortoiliac segment were treated (Table 1). The decision to perform a CERAB technique or implant BeGraft and Solaris stent grafts was left to the operator’s judgment. All operators were qualified consultant vascular surgeons. Other treatment modalities included the kissing-stent technique, the CERAB technique with the use of other covered or uncovered stents, or open surgical procedures (aorto-bifemoral bypass of femoro-femoral bypass).

### 2.2. Patients

All lesions were evaluated with a pre-operative computed tomography angiography (CTA). The degree of calcification was assessed routinely. They were graded on the basis of the amount of calcium deposits visible during pre-operative CTAs and procedure fluoroscopy: (a) mild for <5 mm calcium deposits in the vessel wall, (b) moderate for >5 mm deposits, and (c) severe for deposits filling up the entire vessel diameter.

Preoperative CTAs and procedure angiograms were analyzed with regard to lesion properties (lesion location, lesion length, vessel diameter, degree of stenosis, and calcification) by two investigators (ESN and AR). Interobserver and intraobserver agreement showed kappa values of 0.92/0.87, 0.93/0.95, 0.91/0.92, 0.95/0.94, and 0.96/0.96, respectively.

Demographic data, risk factors, and lesion characteristics are reported in Table 2. A total of 42 patients were included. The mean age was 72 years (range 59–85). Severe claudication (42.9%) was the most common primary indication for intervention, followed by rest pain (28.6%), minor tissue loss (14.3%), and severe tissue loss (14.3%). Hypertension and hyperlipidemia were the two most common risk factors, with 85.7% of patients being hypertensive and 76.2% hyperlipidemic. The most frequent American Society of Anesthesiologists classification was Category III (61.9%). The mean lesion length was 121.24 ± 7.35 mm. Occlusive lesions presented in 90.5% and moderate or severe calcification was usually (81.0%).

### 2.3. Technique

**Figure 1:**
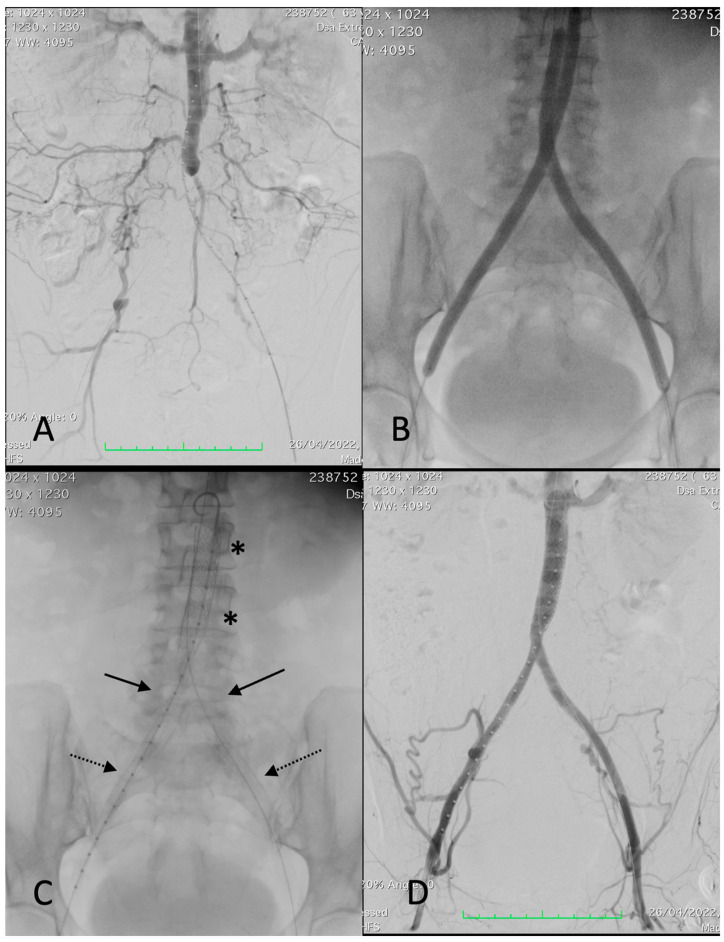
Procedural images of a 67-year-old female patient with left-sided Rutherford 4. (**A**) angiography showing thrombosis of common and external left iliac arteries and critical stenosis of the right common iliac artery with post-stenotic dilatation, extending into the distal aorta. (**B**) Deployment of a 12 × 39 mm BeGraft aortic (asterisks). (**C**) Deployment of two BeGraft Plus 7 × 57 mm stent grafts (arrow) and a Solaris stent graft (6 × 100 mm) at the left external iliac artery (dotted arrow). (**D**) Completion angiography showing a patent reconstruction with a good outflow.

The CERAB technique has been described previously by Goverde et al. [1]. All patients had initial diagnostic angiography of the aorta and both iliac arteries with a standard contrast intra-arterial digital subtraction angiogram to outline the vascular anatomy and define the lesion characteristics (Figure 1). The endovascular procedures were performed by experienced vascular surgeons in a hybrid endovascular suite (Siemens Artis Zeego; Siemens, Munich, Germany). All patients were on aspirin pre-operatively. The procedures were performed under monitored anesthetic care. Percutaneous femoral accesses were used and 9 Fr or 11 Fr sheaths were introduced into one common femoral artery (BeGraft aortic covered stent recommended sheaths were 9 Fr for 12 mm stent diameter and 11 Fr for 14 mm diameter) and another 7 or 8 Fr sheath into the contralateral common femoral artery. Two closure devices (Perclose, Abbott Vascular, Abbott Park, IL, USA) were prepared during the femoral access for the 9 Fr sheaths. Periprocedural anticoagulation with weight-adjusted doses of intravenous heparin was administered. Stenosis and occlusions were passed, either subintimal or endoluminal, using 0.035′′ or 0.018′′ guidewires with the help of diagnostic catheters. After gaining re-entry into the lumen of the aorta, angiography confirmed the proper positioning for those with a subintimal passage. A 12 or 14 mm BeGraft aortic balloon-expandable covered stent (Bentley InnoMed, Hechingen, Germany) was implanted in the distal aorta, approximately 20 mm above the bifurcation. The proximal 2/3 part of the aortic stent was flared with a larger balloon (diameter was selected based on the proximal aortic lumen diameter without significant stenosis, considered the maximum post-dilatation diameter of 20 mm). Subsequently, two BeGraft Plus stent graft (Bentley InnoMed, Hechingen, Germany) balloon-expandable covered stents were deployed simultaneously, proximally in the distal 1/3 of the aortic stent and distally into the common iliac arteries. Their diameter varied from 7 to 8 mm depending on the distal common iliac artery lumen diameter, all without significant stenosis. When required, distal extensions were added, in those cases, Solaris self-expanding stent grafts (Scitech Medical, Goiania, Brasil). Nevertheless, in cases with a patent hypogastric artery and a fully diseased common and external iliac artery, a bare stent was deployed at that level. The sheaths were removed from the common femoral artery and the puncture sites were closed, usually using a closure device (Perclose Proglide, Abbott Vascular, IL, USA) or sutured in cases of open introduction. Technically, there are no anatomic or morphologic lesion boundaries for indication of the CERAB technique. When treating a total chronic occlusion, it was difficult to determine whether the guidewire was advanced intraluminally or subintimally; however, lesions were treated entirely with covered stents. Re-entry to the vascular lumen was always made as distal as possible in the patent aorta. When renal arteries or even visceral arteries were involved in the diseased segment, the use of protective balloons (3 cases) or chimney grafts (no cases) was considered. A concomitant endarterectomy of the femoral artery and/or external iliac artery was performed in cases of multilevel disease. Digital subtraction angiography (DSA) after each procedure recorded the stent implantation sites and runoff vessels to assess diameter improvement and potential complications, (such as dissection, thrombosis, or embolism). All DSA studies were conducted and interpreted by the same examiners (ESN, JAB). Post-intervention dual antiplatelet therapy with aspirin (100 mg/d) and clopidogrel (75 mg/d) was given for at least 4 weeks, with aspirin continued indefinitely thereafter. Statin therapy was also indicated.

### 2.4. Follow-Up

All patients were followed in a post-interventional surveillance program that included clinical evaluation by a consultant vascular surgeon; ankle brachial index (ABI) measurement; and color duplex imaging at 3, 6, and 12 months and every year thereafter. If significant in-stent stenosis recurred, the patient was scheduled for angioplasty, drug-eluting balloon angioplasty, or new stenting. If associated runoff vessel stenosis was detected during the follow-up, the patient was scheduled for endovascular treatment or open repair.

Duplex examination was performed by the same experienced vascular surgeon using a Philips duplex ultrasound system (MyLab 60; Esaote, Genoa, Italy) with a 7.5 MHz linear multifrequency transducer. Stenoses were identified based on a significant peak systolic velocity (PSV) ratio of ≥2.0, a PSV gradient >300 cm/s at the lesion site, a post-stenotic PSV <40 cm/s, or a drop in ABI >0.15. A significant stenosis was defined at angiography as a >50% reduction in vessel diameter based on a ratio of the minimal intrastenotic to prestenotic vessel diameters [6]. 

### 2.5. Definition and Outcomes Measure

Technical success was defined as an improvement in luminal diameter >50% or residual stenoses <30% in keeping with the reporting standards of the Society for Vascular Surgery for the endovascular treatment of lower limb peripheral artery disease [6]. Restenosis was indicated by a 2-fold increase in the PSV gradient at duplex examination, a PSV gradient >300 cm/s at the lesion site, a post-stenotic PSV <40 cm/s, or a drop in ABI >0.15. Device success was defined as correct balloon angioplasty and stent deployment and removal. Procedural success combined device and technical success with the absence of procedure-related complications (dissection, perforation, distal embolism, in situ thrombosis, arteriovenous fistula, or pseudoaneurysm formation) or bailout. 

The primary outcome of this analysis was primary patency at the 12-month follow-up, defined as the absence of binary restenosis or reocclusion on duplex ultrasound examination without repeat target lesion interventions. Primary-assisted patency, secondary patency, mortality, amputation rate, clinical status, and ABI measurements, were also analyzed. Primary assisted patency was defined as a patent aotoiliac segment that underwent further intervention within the inflow, treated vessel segment, or outflow of the treated vessel segment to improve patency. Secondary patency was defined as requiring a secondary intervention to restore patency after the occlusion of the treated segment. Secondary interventions include PTA, drug-eluting balloon PTA (DEB-PTA), additional stent placement, or surgical bypass based on clinical deterioration. Major and minor complications were registered up to 30 days after the procedure. Complications leading to transient impairment were scored as minor. Complications leading to permanent damage or death were scored as major. The limb salvage rate was defined as all patients without above-the-knee amputations. 

### 2.6. Statistical Analysis

Continuous variables were reported as mean and standard deviation (SD). Follow-up duration was presented as median and range. Categorical variables were reported as absolute values and proportions (%). In all patients, the mean ABI of both legs was calculated to compare pre- and post-procedural ABI with paired *t*-tests. Patency and limb salvage rates were estimated using the Kaplan–Meier survival analysis on a per-patient basis. A significance level of 5% was used. All hypothesis tests were two sided and *p* < 0.05 was considered significant. Univariate and multivariate Cox regression analyses were performed to identify risk factors associated with vessel patency. All calculations were performed with the SPSS statistical software package (version 27; IBM Corporation, Somers, NY, USA).

## 3. Results

During the study period, a total of 42 patients (32 male and 10 female) were treated with the CERAB technique using the combination of BeGraft and Solaris stent grafts. In the majority of cases, five stents (one BeGraft aortic, two BeGraft Plus, and two Solaris) were implanted (57.1%); however, in eight (19.0%) cases, four stents were used; in six (14.3%), seven stents were implanted; and in four (9.5%) cases, eight stents were required. Several patients required hybrid procedures to revascularize the limbs. The associated open surgeries performed were fourteen femoral thromboendarterectomies (33.3%), ten digital amputations (23.8%), and six transmetatarsal amputations (14.3%). The lesion localizations are described in Table 3.

The procedure success was 100%. The median patient follow-up time was 14 months (3–36). Conversion to open surgery was not necessary and there were no in-hospital deaths. The median hospital length of stay was two days (range one to eleven). The in-hospital adverse events (23.8% minor complications) were limited to three puncture-site pseudoaneurysms, which were managed by thrombin injection, and seven minor access site hematomas. One surgical wound dehiscence was observed in a patient treated with femoral thromboendarterectomy. One patient died at a 10-month follow-up due to lung cancer. No complications in the form of acute kidney injury secondary to IV contrast or renal artery dissection were observed. The mean preoperative ABI increased from 0.37 ± 0.19 before intervention to 0.71 ± 1.23 post-operatively at 12 months (*p* = 0.037). The limb salvage rate was 100%.

**Figure 2:**
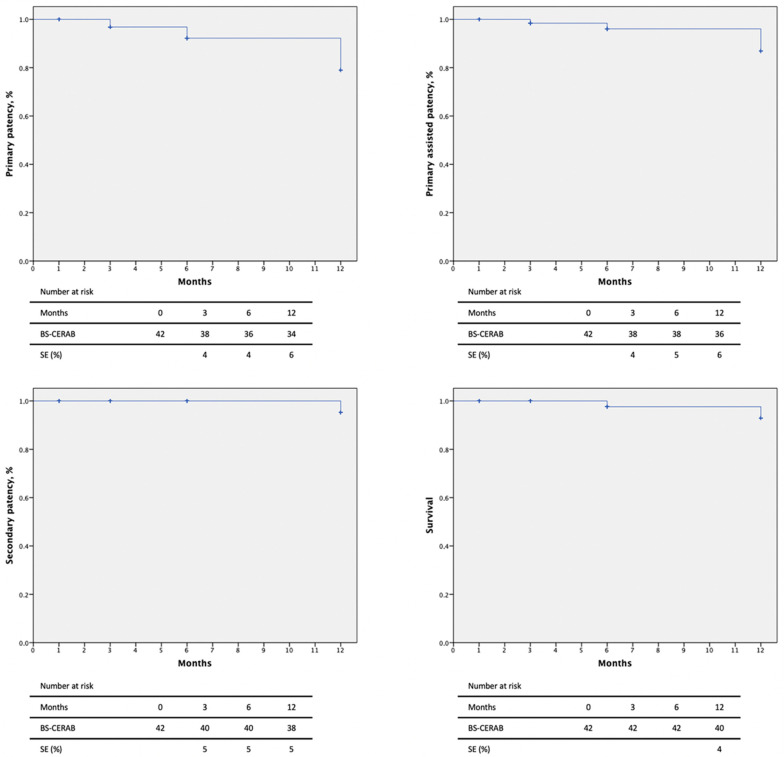
Patency rates and mortality of 42 patients with severe aortoiliac atherosclerotic disease treated with the CERAB technique using the combination of BeGraft and Solaris stent grafts.

Primary patency rates at 3, 6, and 12 months were 90.5%, 85.7%, and 81.0% and primary assisted patency rates were 90.5%, 90.5%, and 85.7%, respectively, by Kaplan–Meier estimates (Figure 2). Two intrastent stenoses were discovered at 3 and 6-month follow-ups and were treated by a DEB-PTA, with technical and clinical success (improvement in luminal diameter >50% or residual stenoses <30%). Secondary patency was 95.2% at 3 and 6 months and 90.5% at the 12-month follow-up. One patient suffered an iliac axis thrombosis, requiring a femoro-femoral bypass. 

Patients with active cancer (*p* = 0.023, OR 2.12 95%CI 1.14–3.25) were a positive predictor for restenosis in this cohort. Primary patency was not affected by age (*p* = 0.238), gender (*p* = 0.167), presence of occlusion (*p* = 0.130), calcification degree (*p* = 0.231), or other cardiovascular risk factors as these variables did not constitute risk factors for stent restenosis.

## 4. Discussion

This study describes our initial experience with primary covered stenting using the CERAB technique with BeGraft and Solaris stent grafts for treating symptomatic patients with aortoiliac atherosclerotic lesions. This series reflects a real-world experience and, despite the challenging study population, these patients may be treated successfully with excellent results, satisfactory patency rates, and few reinterventions. 

The possible endovascular strategies for the treatment of aortoiliac obstructive disease involving the aortic bifurcation are the kissing stents technique, the use of low-profile endografts, or the CERAB technique [7]. Covered stents are chosen for CERAB for several reasons. Moreover, the COBEST trial showed the superiority of covered stents for TASC-II C and D lesions above bare stents regarding patency rate and survival benefit after a 5-year follow-up [8]; however, the benefits of the covered stents seem mainly to be manifested in larger, more complex lesions. The differences between balloon-expandable and self-expanding platforms used for the treatment of aortoiliac lesions are technical. There are currently no robust studies demonstrating any benefits for balloon-expandable versus self-expanding stents for iliac lesions [9,10]. Obviously, comparative trials would be needed to select the right technique for the right patient. The CERAB technique was designed to achieve a more optimal anatomical and physiological reconstruction in order to improve the results of the endovascular treatment of the aortic bifurcation [11,12]. The use of covered stents may reduce the incidence of complications during the endovascular treatment of aortoiliac steno-occlusive lesions as they showed fewer complications and better patency rates than bare metal stents. The total covering of wall thrombus and ulcered plaques (thus preventing distal embolization) constitutes a great advantage of the covered stents. On the other hand, covered stents serve as a mechanical barrier. In fact, when bare metal stents are implanted, these macrophages are allowed to infiltrate the endothelium, initiate another pro-inflammatory process (which may involve cytokines), and, therefore, the growth of neointimal hyperplasia and subsequent restenosis. Additionally, the decreased risk of iliac rupture in patients treated with covered stents may lead to improved dilation with the use of higher inflation pressures.

In a study published in 2015, 103 patients suffering from obstructive lesions of the aortic bifurcation were treated with CERAB. Primary patency at 1 year was 87.3% and it was 82.3% at 2 years while secondary patency rose to 95.0% at 1 and 2 years [13]. Additionally, 88 patients (85.4%) presented TASC-II D lesions and the technical success was 95.1%. Taeymans et al. [14] published, in 2018, the results of the CERAB technique for the treatment of 130 patients with extensive aortoiliac occlusive disease. TASC-II D lesions were mainly treated (89%) and the technical success rate was 97%. Primary patency was 86%, 84%, and 82% at the 1-, 2-, and 3-year follow-ups, respectively. These results are comparable to those in our series (primary patency 81.0% at 1 year). More recently, in 2021, Saratzis et al. [15], reported the results of a UK multicenter study that includes 116 patients that underwent CERAB. Freedom from TLR at the 1-year follow-up was 94% and primary patency, assisted primary patency, and secondary patency were 88%, 95%, and 98%, respectively. Nowadays, CERAB results after 5 years of follow-up have been published. Rouwenhorst et al. [16], reported on 160 patients with extensive aortoiliac occlusive disease consecutive electively treated by CERAB; in total, 75.6% presented claudication and 83.1% had TASC-II D lesions. The 5-year primary patency, primary assisted patency, and secondary patency rates were 77.5%, 88.1%, and 95.0%, respectively.

Although the systematic review and meta-analysis published by Salem et al. [3] in 2021 showed that open bypass procedures for the management of extensive aortoiliac disease presented better primary patency than endovascular techniques after 1 and 3 years of follow-up, the secondary patency was comparable in both groups. These findings may be explained by the computational analysis published during 2023 comparing the open surgical repair of aortoiliac occlusive diseases, such as aorto-bifemoral bypass and axilo-bifemoral bypass, with endovascular options such, as kissing stent and CERAB, has been published [17]. Its main conclusion was the favorable haemodynamic performance of the aorto-bifemoral bypass compared with the rest of the possible open or endovascular configurations. Those conclusions could explain the lower primary patency rates of the axilo-bifemoral bypass and the endovascular techniques compared to the aorto-bifemoral bypass [18]. Groot Jebbink et al. [2] compared the flow perturbations (measured by peak systolic velocity, end-systolic velocity, peak diastolic velocity, and wall shear stress) between a bare metal kissing stent, covered kissing stent, and CERAB for the endovascular reconstruction of the aortic bifurcation and concluded that the CERAB configuration was the most unimpaired physiologic reconstruction.

The only identified risk factor for restenosis was active cancer (*p* = 0.023, OR 2.12 95%CI 1.14–3.25). The pathogenesis of arterial thrombosis in cancer is not totally known. Endothelial damage and platelet activation could promote thrombosis by exposing procoagulant molecules within the atherosclerotic plaque and the surface of the implanted stents.

The past decade has seen remarkable progress in technology and endovascular procedures, particularly in dealing with complex atherosclerotic lesions like aortoiliac chronic total occlusions. Apart from the primary stenting of the iliac arteries, the “kissing stents” configuration is one of the more usual ways to involve the stenting of the aortic bifurcation. As with any new endovascular technique, the CERAB technique has several limitations. Compared with the traditional kissing stents technique, the CERAB with BeGraft and Solaris stent grafts requires multiple access sites (on several occasions femoral and humeral) and a larger profile introducer sheath (9 F–14 F), not to mention that the procedure is probably more expensive. Finally, the flaring technique of the proximal aortic stent commonly used during CERAB is still out of the instruction for use (IFU) for BeGraft-covered stents. Placing a covered stent in the abdominal aorta also has a higher potential for coverage of the inferior mesenteric artery and collateral vessels. In our experience, no spinal cord ischemia or visceral ischemic events occurred. Our initial experience with CERAB procedures showed low procedural-related complications, with 100% procedure success and three puncture-site pseudoaneurysms, which were managed by thrombin injection, and seven minor access site hematomas. No complications related to the stent’s implantation were observed. Another issue to take into account is that covered stents are more expensive than bare metal stents and, therefore, the cost-effectiveness of CERAB procedures remains unclear.

The relative paucity of endovascular devices specifically designed for aortoiliac occlusive disease has led vascular specialists to pay particular attention to the potential flow perturbations and thrombus formation determined by the geometry of the reconstruction [15,18]. There has been an evolution in stent design since this original publication and several balloon-covered stents have been used at the aortic bifurcation level for this technique as Advanta V12 (Atrium Medical Corporation, Hudson, NH, USA), BeGraft (Bentley InnoMed, Hechingen, Germany) [19], or Viabahn VBX (WL Gore & Associates, Flagstaff, AZ, USA) [20]. We preferred the combination of BeGraft Aortic and BeGraft Plus stent grafts because they have higher circumferential radial force compared to other balloon-expandable covered stents or even with the BeGraft Stent Graft. During 2023, Kruszyna et al. [21] reported 120 patients with extensive aortoiliac occlusive disease being treated using BeGraft balloon-expandable covered stents. Primary patency, secondary patency, and freedom from TLR at the 12-month follow-up were 94.5%, 97.3%, and 93.5%, respectively. The overall procedural complication rate was low (11.7%) and the ABI increased significantly (*p* < 0.05). When distal extensions to common or external iliac arteries were required, several covered or uncovered stents were used, according to the literature; nevertheless, in those cases, we chose the Solaris self-expanding stent graft (Scitech Medical, Goiania, Brasil). Solaris is a flexible self-expandible endograft composed of a thin, multidirectional, durable, electrospinning polytetrafluoroethylene membrane encapsulating a nitinol stent structure. The device’s design provided high flexibility without compromising the requirement length, balanced radial force, and low shortening rate [22].

This study presented several limitations. The data reflect a multicenter (two centers) experience; thus, potential confounding variables are possible, for example, selection bias and disproportional distribution in data collection that may not extrapolate to other institutions. This study had a limited group size and lacked randomization, which may have influenced the results. Another limitation is the lack of judgment integrity by an independent core laboratory and, obviously, this study still requires more follow-ups for definite results. However, this cohort is the largest in the literature with the combination of BeGraft and Solaris stent grafts for carrying out a CERAB technique and provides a real-world experience. 

## 5. Conclusions

This 1-year long experience shows that the combination of BeGraft and Solaris stent grafts may allow a feasible and safe endovascular reconstruction of the aortic bifurcation in patients with severe steno-occlusive atherosclerotic disease and a high incidence of cardiovascular risk factors. The conformability and flexibility in the iliac arteries allow an excellent final configuration and optimal patency.

## Figures and Tables

**Table 1:** Endovascular procedures performed during the study period between January 2020 and December 2023 (48 months).

	N = 271	%
Rutherford class		
2 3 4 5 6	103 65 43 32 28	38.0% 24.0% 15.9% 11.8% 10.3%
TASC II		
Stage A Stage B Stage C Stage D	63 82 69 57	23.2% 30.3% 25.5% 21.0%
Occlusion lesion	165	60.9%
Length of treated lesion (mm mean, SD)	81.16 ± 11.63	
Severe/moderate calcification	158	58.3%
Runoff vessel lesions		
Common femoral arteries Superficial femoral arteries	79 167	29.2% 61.6%
Concomitant open procedures		
Femoral endarterectomy Femoro-femoral bypass Femoro-popliteal bypass	77 31 38	28.4% 11.4% 14.0%

**Table 2:** Baseline characteristics and perioperative details of patients undergoing CERAB and PTA/covered stenting with BeGraft and Solaris stent grafts. COPD: chronic obstructive pulmonary disease; CHF congestive heart failure; CAD: coronary artery disease. ABI: ankle-brachial index.

	*n*	%
Age (years mean, SD)	72.3 ± 9.35	
Gender (male)	32	76.2%
Hypertension	36	85.7%
Hyperlipidemia	32	76.2%
Diabetes mellitus	18	42.9%
History of smoking	30	71.4%
COPD	8	19.0%
CHF	4	9.5%
CAD	10	23.8%
Renal insufficiency	6	14.3%
Cerebrovascular disease	2	4.8%
History of cancer	12	28.6%
Active cancer	8	19.0%
ASA classification		
II III IV	10 26 6	23.8% 61.9% 14.3%
Rutherford class		
3 4 5 6	18 12 6 6	42.9% 28.6% 14.3% 14.3%
Aspirin	26	6.2%
Clopidogrel	8	19.0%
Anticoagulation	10	23.8%
TASC II stage D	42	100.0%
Occlusion lesion	38	90.5%
Length of treated lesion (mm mean, SD)	121.24 ± 7.35	
Severe/moderate calcification	34	81.0%
Runoff vessel lesions		
Common femoral arteries Superficial femoral arteries	14 32	33.3% 76.2%
Preoperative ABI	0.37 ± 0.19	

**Table 3:** Details of the aortoiliac arteries treated.

	Open	Stenosis	Occluded			
Aorta	0 (0%)	30 (71.4%)	12 (28.5%)			
Iliac arteries	Right			Left		
	Open	Stenosis	Occluded	Open	Stenosis	Occluded
Common iliac artery	0 (0%)	10 (23.8%)	32 (76.2%)	0 (0%)	14 (33.3%)	28 (66.6%)
External iliac artery	8 (19.0%)	22 (52.4%)	12 (28.6%)	14 (33.3%)	20 (47.6%)	8 (19.0%)
Internal iliac artery	6 (14.3%)	30 (71.4%)	6 (14.3%)	12 (28.6%)	16 (38.1%)	14 (33.3%)

## Data Availability

The data underlying this article will be shared upon reasonable request from the corresponding author.

## Supplementary Materials

The following supporting information can be downloaded at: https://www.mdpi.com/article/10.3390/jcm13071925/s1, Table S1: STROBE Statement—Checklist of items that should be included in reports of cohort studies.
